# Copper Nanoparticles and Copper Sulphate Induced Cytotoxicity in Hepatocyte Primary Cultures of *Epinephelus coioides*

**DOI:** 10.1371/journal.pone.0149484

**Published:** 2016-02-18

**Authors:** Tao Wang, Xiaoyan Chen, Xiaohua Long, Zhaopu Liu, Shaohua Yan

**Affiliations:** Jiangsu Provincial Key Laboratory of Marine Biology, College of Resources and Environmental Sciences, Nanjing Agricultural University, Nanjing, 210095, P.R. China; University of Pecs Medical School, HUNGARY

## Abstract

Copper nanoparticles (Cu-NPs) were widely used in various industrial and commercial applications. The aim of this study was to analyze the cytotoxicity of Cu-NPs on primary hepatocytes of *E*.*coioides* compared with copper sulphate (CuSO_4_). Cultured cells were exposed to 0 or 2.4 mg Cu L^-1^ as CuSO_4_or Cu-NPs for 24-h. Results showed either form of Cu caused a dramatic loss in cell viability, more so in the CuSO_4_ than Cu-NPs treatment. Compared to control, either CuSO_4_ or Cu-NPs significantly increased reactive oxygen species(ROS) and malondialdehyde(MDA) concentration in hepatocytes by overwhelming total superoxide dismutase (T-SOD) activity, catalase(CAT) activity and glutathione(GSH) concentration. In addition, the antioxidative-related genes [*SOD (Cu/Zn)*, *SOD (Mn)*, *CAT*, *GPx4*] were also down-regulated. The apoptosis and necrosis percentage was significantly higher after the CuSO_4_ or Cu-NPs treatment than the control. The apoptosis was induced by the increased cytochrome c concentration in the cytosol and elevated caspase-3, caspase-8 and caspase-9 activities. Additionally, the apoptosis-related genes (*p53*, *p38β* and *TNF-α*) and protein (p53 protein) were up-regulated after the CuSO_4_ or Cu-NPs treatment, with CuSO_4_ exposure having a greater effect than Cu-NPs. In conclusion, Cu-NPs had similar types of toxic effects as CuSO_4_ on primary hepatocytes of *E*.*coioides*, but toxicity of CuSO_4_ was more severe than that of Cu-NPs.

## Introduction

Owing to their unique chemical and physical properties that emerge at the nanoscale, engineered copper nanoparticles(Cu-NPs) are found in a broad range of industrial and scientific applications as well as in consumer products [1,2].They are also used in environmental remediation for the removal of organic pollutants or as a versatile biocide [3].Even though true global production volumes of Cu-NPs are presently unknown, their broad use is highly likely going to result in a release into aquatic systems and the contact with non-target, aquatic organisms[2,4].In the last decade, concerns were raised about the effects of metal nanoparticles on fish, including sublethal effects of nanoparticles on the different body systems of fish [5–7]. However, most studies to understand the toxicity of Cu-NPs were performed in whole fish. To our knowledge, the understanding of Cu-NPs toxicity to fish cells is limited, and no study reported toxicity of Cu-NPs on primary hepatocytes of *E*.*coioides*.

In aquaculture production, CuSO_4_ is used to control diseases and algae [8]. Toxicity of CuSO_4_ to fishes is relatively well known, including tissue injuries[9,10], osmoregulatory disturbances [11,12], and oxidative stress [9,13,14]. However, so far, no study has investigated possible mechanisms of CuSO_4_ toxicity on hepatocytes of *E*.*coioides*.

*E*.*coioides*, a protogynous hermaphroditic fish, is a major farmed marine fish in China and Southeast Asian counties[15].Currently, *E*.*coioides* were mainly cultured in floating net cages and earthen ponds in the natural environment that can easily be affected by environmental pollution[16].The discharge of Cu-NPs or CuSO_4_in the aquatic environment is inevitable [10].A deeper understanding of the toxic effects of Cu-NPs or CuSO_4_ to *E*.*coioides* might help in development of guidelines to fish culture, and also to human food safety.

Previously, we studied the toxic effects of Cu-NPs on juvenile *E*.*coioides*(cultured in seawater) compared with CuSO_4_, and found that Cu from CuSO_4_ was more toxic to liver than the equal concentration of Cu-NPs [7]. In the present study, we investigated the toxic effects of Cu-NPs and CuSO_4_ on hepatocyte primary cultures of *E*.*coioides*. The aims of this study were to find whether liver toxicity *in vivo* can be replicated in hepatocyte cultures, and to explore the mechanisms of Cu-NPs and CuSO_4_ toxicity to *E*.*coioides* at the cellular level.

## Materials and Methods

### Ethics statement

All experimental protocols were approved by the Institutional Animal Care and Use Committee of Nanjing Agricultural University (Nanjing, China). To collect tissues, fish were euthanized (MS-222 at 10 mg L^-1^) according to the Guide for the Care and Use of Laboratory Animals in China.

### Nanoparticle characterization

The Cu-NPs were purchased from Shanghai Aladdin Co., Ltd. China, with an average particle diameter of 20±10nm and 99.9% purity (manufacturer’s specification). Cu-NPs suspension containing 1.0 g Cu L^-1^ was prepared by dispersing nanoparticles in ultrapure water(Millipore, ion free and unbuffered), sonicated for 30 min and stirred for 1 h at room temperature to increase dispersion before use[10,17]. Copper ion stock solution (1.0 g Cu L^-1^) was prepared by dissolving 3.929 g CuSO_4_·5H_2_O in 1 L of ultrapure water.

As in our previous study [18], the particle size was characterized using transmission electron microscopy (TEM, JEOL JEM-2100, Japan) and nanoparticle tracking analysis (NTA, NanoSight LM_10_). For TEM analysis, Cu-NPs were diluted in ultrapure water (Millipore, ion free and unbuffered) and sonicated 30 min to keep the particles in solution and avoid aggregation [19,20]. The primary range of particle sizes was determined from micrographs through analysis of 50 NPs selected randomly. Additionally, particle size distribution in the cell culture medium[DMEM/F12 medium in the presence of 15% (v/v) serum, 100 IU mL^-1^penicillin and 100 IU mL^-1^ streptomycin] were measured by NTA in 20 mg Cu L^-1^ to give sufficient particle tracks (>100 tracks per sample)[21].

### Primary hepatocytes from *E*.*coioides*

Hepatocytes culture was carried out as previously described[22]. Briefly, liver was obtained from healthy juvenile *E*.*coioides* and washed several times in ice-cold phosphate-buffered saline (PBS) containing 100 IU mL^-1^ penicillin and 100 IU mL^-1^ streptomycin (Sigma Chemical Company, St. Louis, MO), then the liver was cut into small pieces (about 1 mm), placed into 25-cm^2^ tissue culture flasks and cultured in fresh Dulbecco modified Eagle F-12 medium (DMEM) in the presence of 15% (v/v) serum and 100 IU mL^-1^ of each penicillin and streptomycin at 28°C in an incubator with 5% (v/v) CO_2_; the medium was replaced every 3 days. After 1 week, cells grew out of the explants, and the explants were removed. Upon reaching confluency, cells were harvested in 0.25% (w/v) trypsin-EDTA, suspended at a density of 1×10^6^ cells mL^-1^, and counted using 0.4% (w/v) trypan-blue and an inverted microscope (TE 2000 Microscope, Nikon, Japan) to confirm that cell viability exceeded 90% for subsequent experimentation.

### Hepatocyte sub-culturing and treatment

Cell suspensions were seeded onto 6-well or 96-well plates depending on the experiments, incubated at 28°C with 5% (v/v) CO_2_.The medium (as above) was replaced every 3 d until 80–90% confluent, at which time cells were transferred into culture medium containing 0 or 2.4 mg Cu L^-1^ as CuSO_4_ or Cu-NPs for 24 h. Three replicates were performed for each treatment. The concentration of 2.4 mg Cu L^-1^ Cu was chosen because it represents half of 24-h LC_50_ concentration of Cu-NPs for juvenile *E*.*coioides*. In addition, this concentration reflects point-source environmental pollution (e.g. some areas with intensive agricultural and mining activities, manufacturing industries and municipal waste depositions) [23].After exposure, cells were harvested in 0.25%(w/v) trypsin-EDTA at room temperature for cellular biochemical assays.

### Cytotoxicity assays

Cytotoxic responses of *E*.*coioides* hepatocytesin CuSO_4_ or Cu-NPs were measured by the MTT [3-(4,5-dimethyl-2-thiazolyl)-2,5-diphenyl-2-H-tetrazoliumbromide] and LDH (lactate dehydrogenase) leakage assays.

### MTT assay

Viability of hepatocytes after exposure to CuSO_4_ or Cu-NPs was assessed by MTT assay as described by Ahamed et al.[24] with some modifications. The MTT assay assesses the mitochondrial function by measuring ability of viable cells to reduce MTT into blue formazan product. In brief, suspension of primary cell cultures (1× 10^5^ cells mL^-1^) was seeded in 6-well plates, incubated at 28 °C with 5% (v/v) CO_2_ until 80–90% confluent, then exposed to control (0) or 2.4 mg Cu L^-1^ as CuSO_4_ or Cu-NPs for 24 h. At the end of exposure, solution of MTT (0.5 mg mL^-1^) was added and cells were incubated under normal culture conditions for 4 h. The resulting formazan crystals were dissolved in isopropanol acidified with 0.04 N HCl. Further, a 200-μL aliquot of supernatant was transferred to clean wells of a 96-well plate, and absorbance was measured at 570 nm by using a microplate reader (Bio-Rad, USA).

### LDH leakage assay

The assay was carried out using an LDH cytotoxicity assay kit (Beyotime Institute of Biotechnology, Haimen, Jiangsu, China). In brief, 1 ×10^4^ cells/well were seeded in 96-well plates at a final volume of 200μL culture medium/well, incubated at 28 °C with 5% (v/v) CO_2_ until 80–90% confluent, then exposed to control (0) or2.4 mg Cu L^-1^ as CuSO_4_ or Cu-NPs for 24 h. At the end of treatment, LDH concentration in the media and the cells was quantified and compared to the control values using a microplate reader (Bio-Rad, USA) at 340 nm according to the manufacturer’s protocol.

### Reactive oxygen species (ROS) generation

ROS generation was measured using 2,7-dichlorofluoresceindiacetate (DCFH-DA) as described by Xu et al. [22] with some modifications. In brief, 500 μL of hepatocytes (1 × 10^7^ cells mL^-1^) were collected after CuSO_4_ or Cu-NPs exposure, then incubated in PBS buffer with10 μmol L^-1^ DCFH-DA at 28°C in the dark for 1 h, and the fluorescence intensity was measured at 485 nm excitation and 520 nm emission using a microplate reader (Bio-Rad, USA). The values were expressed as a percent of fluorescence intensity relative to control wells.

### Oxidative stress detection

After CuSO_4_ or Cu-NPs exposure, hepatocytes (1×10^7^ cells mL^-1^) were collected, homogenized with a glass grinder, and centrifuged at 800×*g* at 4°C for 10 min; the supernatant was used for oxidative stress analysis. Activities of catalase (CAT) and total superoxide dismutase (T-SOD) and concentrations of malondialdehyde (MDA) and glutathione (GSH) were detected using kits (Nanjing Jiancheng Bioengineering Research Institute, Nanjing, China) according to the manufacturer’s instructions [7,22]. CAT activity was determined based on ammonium molybdate spectrophotometry. T-SOD activity was determined based on the WST-1 (water-soluble tetrazolium salt) method. The MDA concentration was determined via modified NBT (nitroblue tetrazolium) method [25]. GSH was determined based on the recycling reaction of GSH with DNTB (5,5′-dithios-(2-nitrobenzoic acid)) in the presence of excess glutathione reductase. The protein content was determined using the Bradford’s method [26].

### Cell apoptosis and necrosis detection

According to Cui et al. [27], apoptotisis and necrosis were quantified by Annexin V-FITC/PI apoptosis detection kit (Nanjing Key-gen Biotech. Co., Ltd. Nanjing, China). Briefly, cells were collected after CuSO_4_ or Cu-NPs exposure (5×10^5^ cells mL^-1^) and washed with PBS. The cells were resuspended in the Annexin V-FITC staining reagent and fixed at 28°C for 15 min. Then the cells were washed and resuspended in the PI staining reagent. Staining was stable at 4°C for 30 min[27,28]. Samples were then analyzed by flow cytometry (Becton Dickinson, San Jose, CA).

### Nuclear ultrastructure observation

The collected cells (1×10^7^ cells mL^-1^) were fixed with PBS containing 2.5% (v/v) glutaraldehyde followed by treatment with PBS containing 1% (v/v) osmium tetroxide (OsO_4_). The samples were dehydrated in a graded series of alcohol (30%, 50%, 70%, 80%, 90%, 95% and 100%, v/v) for 15 min at each concentration, embedded in epoxy resin Epon812, and sectioned(60 nm) using an ultra-microtome (LKB Nova, Bromma, Sweden). Ultrathin sections were stained with 4% (w/v) uranyl acetate, as well as lead citrate, and observed using an H-7650 transmission electron microscope (Hitachi High-Technologies Co., Japan).

### Cytochrome c concentration

After exposure, hepatocytes (1×10^7^ cells mL^-1^) were harvested and centrifuged at 800×*g* at 4°C for 10 min; the pellets were used for cytochrome c detection. According to Xu et al. [22], cytochrome c was analyzed by isolation of mitochondrial and cytosolic proteins using amitochondria/cytosol fractionation kit (Nanjing Jiancheng Bioengineering Research Institute, Nanjing, China). The details were described in our previous study[18]. Cytochrome c concentration was measured using enzyme-linked immunosorbent kit (ELISA) (Nanjing Jiancheng Bioengineering Research Institute, Nanjing, China), according to the manufacturer’s protocol, with the optical density (OD) of each well determined using an ELISA reader at 450 nm.

### Caspases

Activities of caspase-3, caspase-8 and caspase-9 were measured using commercial kits (Nanjing Jiancheng Bioengineering Research Institute, Nanjing, China) according to the method of Jia et al. [14]. Briefly, cells (1×10^7^ cells mL^-1^) were collected by centrifugation (800×*g*, 4°C, 10 min) after CuSO_4_ or Cu-NPs exposure and washed twice with PBS, and then homogenized in lysis buffer. The lysates were centrifuged at 10,000×*g* at 4°C for 15 min, and the supernatants were incubated in the dark at 28°C for 4 h with 5 μL (0.2 mM) of each Ac-DEVD-pNA (acetyl-Asp-Glu-Val-Asp p-nitroanilide), Ac-IETD-pNA (acetyl-Ile-Glu-Thr-Asp p-nitroanilide), and Ac-LEHD-pNA (acetyl-Leu-Glu-His-Asp p-nitroanilide) as the substrates for caspase-3,caspase-8 and caspase-9, respectively. The absorbance at 405 nm was read using a microplate reader (Bio-Rad, USA). The reaction with lysis buffer instead of test samples was used as the negative control. The caspase-3, caspase-8 and caspase-9 activities were calculated as OD (treatment)/OD (negative control).

### Gene expression and protein abundance

#### Gene expression

After exposure, cells (1×10^7^ cells mL^-1^) were harvested and centrifuged at 800 ×*g* at 4°C for 10 min and washed twice with PBS; the pellets were used for gene expression and protein abundance analyses. Total RNA was extracted from hepatocytes using RNAiso reagent(Takara Co. Ltd, Japan) according to the manufacturer's instructions. Concentration of extracted RNA was determined using a Nanodrop1000 (Thermo Fisher Scientific, USA), and the integrity of RNA was visualized on 1.0% (w/v) formaldehyde denaturing agarose gel.

The cDNA was synthesised using PrimeScript RT-PCR Kit (Takara Co. Ltd, Japan) according to the manufacturer's instructions. To adjust for the quantity of input cDNA, the house-keeping gene β-actin was used as an internal control. Primers (Table 1) for RT-PCR were designed with reference to the known sequences of *E*.*coioides* in GenBank.

**Table 1:** Nucleotide sequences of the primers used to assay gene expression by real-time PCR.

Target gene	GenBank accession no.	Forward (5’-3’)	Reverse (5’-3’)
β-actin	AY510710	GGCTACTCCTTCACCACCACA	GGGCAACGGAACCTCTCAT
SOD (Cu/Zn)	AY735008	TGGAGAGACCAGTGGGACCGT	GCAGTCACATTTCCCAGGTC
SOD (Mn)	AY735007	CGGCTCCTTCCAGAAGATGA	CTCCCAGTTGATGACATTCCAGAT
CAT	AY735009	GGCGTTTGGTTACTTTGAGGT	AGAAGCGGACAGCAATAGGTG
GPx4	HQ441085	ACATCCTTGCCTTCCCTTC	CCCGTTCACATCGTCCTTA
p53	HM622380	CGCAACAGGCTTCAATCGT	GAAGCATCAGAGGCGAAGA
p38β	JN408833	CAGAGACCTCAAGCCAAGTAATGT	TCGATGTAGTCAGTTCCAGGAAAG
TNF-α	FJ009049	CCTGGTGATGTGGAGATG	GTCCGACTTGATTAGTGCTT

RT-PCR was performed by SYBR Premix EX Taq (Takara Co. Ltd, Japan) using an ABI 7500 Real Time PCR System (Applied Biosystems, Foster City, CA, USA). An aliquot of 2.0 μL of template cDNA was added to the final volume of 20μL of reaction mixture. An RT-PCR protocol consisted of initial denaturation at 95°Cfor 30 s, followed by 40 cycles of 95°C for 3 s each, and annealing at 60°C for 34 s. The dissociation stage included 95°C for 15 s, 60°C for 1 min and 95°C for 15 s. We calculated the relative quantity of the target gene transcripts with a chosen reference gene transcript (β-actin) using the 2^−ΔΔCT^ method [29].

#### Protein abundance

Western blot was used for p53 protein abundance analysis according to the method of Ahamed et al.[24] with some modifications. Cells were lysed in RIPA buffer [1× TBS(0.5 M Tris-HCl and 1.5 M NaCl) pH 7.4, 1% (v/v) NP-40, 0.5% (w/v) sodium deoxycholate, 0.1% (w/v) SDS, 0.004% (w/v) sodium azide] supplemented with DL-dithiothreitol(DTT) and proteinase inhibitors. The cellular lysates were clarified by centrifugation at 15,000× *g* at 4°C for 10 min. Aliquots of the protein extracts were resolved on SDS-PAGE gels, and transferred to nitrocellulose (NC) membranes. Membranes were blocked with 5%(w/v) non-fat dry milk in PBS buffer containing 0.05% (v/v) Tween20, and incubated with rabbit anti-*E*.*coioides* p53 and β-actin monoclonal antibodies (1: 800 dilution) (Abcam, UK) [30]. Specific protein signals were detected by incubating membranes with peroxidase-conjugated anti-rabbit antibodies (1: 2000 dilution) and visualizing with a chemiluminescence reagent (Pierce Biotechnology,Rockford, IL).

### Statistical analysis

Data were analyzed by one-way analysis of variance (ANOVA) using SPASS (19.0). Tukeys’ test was used to compare differences among treatments (*p<*0.05). Data were tested for normality using the Kolmogorov-Smirnov test and for homogeneity of variances by Levene’s test; when necessary, the data were log-transformed [31]. All data were expressed as means ± standard deviation (SD).

## Results

### Characteristics of Cu-NPs used in this study

**Fig 1:**
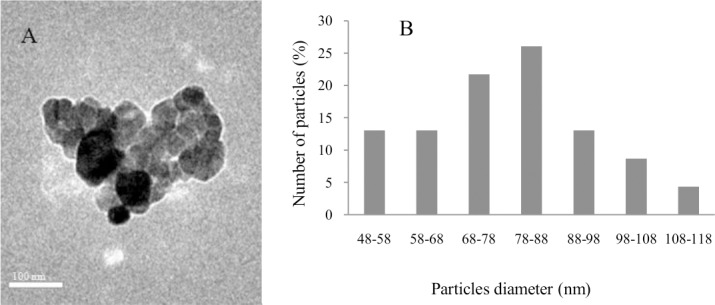
Characteristics of Cu-NPs used in this study. Transmission electron microscopy (TEM) image, scale bar = 100 nm; (B) size distribution of Cu-NPs, with measurements obtained from the TEM images.

The Cu-NPs observed by TEM were spherical in shape and aggregated (Fig 1A), with a mean primary particle diameter of 80±32 nm (mean ±SD, n = 50) (Fig 1B). Using NTA, the average diameter of Cu-NPs in cell culture medium was determined to be 100 ±35 nm.

### Cytotoxicity

**Fig 2:**
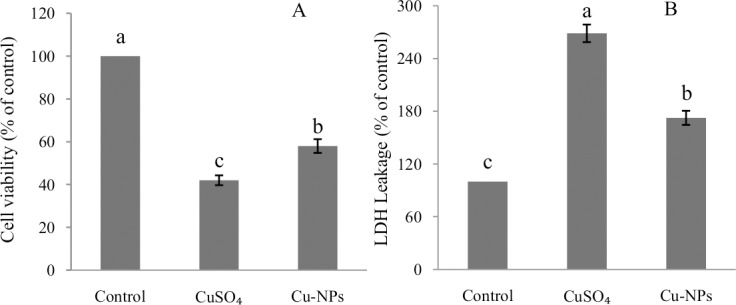
Cell viability and lactate dehydrogenase (LDH) leakage in primary hepatocytes of juvenile *E*.*coioides* after CuSO_4_ or Cu-NPs exposure. Data are means ±SD (n = 3). Significant differences (*p <*0.05) among treatments were indicated by different letters.

Fig 2 represents cytotoxicity induced by Cu from CuSO_4_ or Cu-NPs in the primary hepatocytes of *E*.*coioides*. After exposure, cell viability of hepatocytes significantly decreased compared to control (*p*<0.05), but more so in the CuSO_4_ than Cu-NPs treatment. However, the CuSO_4_ treatment resulted in a significantly higher percentage of LDH leakage than Cu-NPs (*p*<0.05).

### ROS production

**Fig 3:**
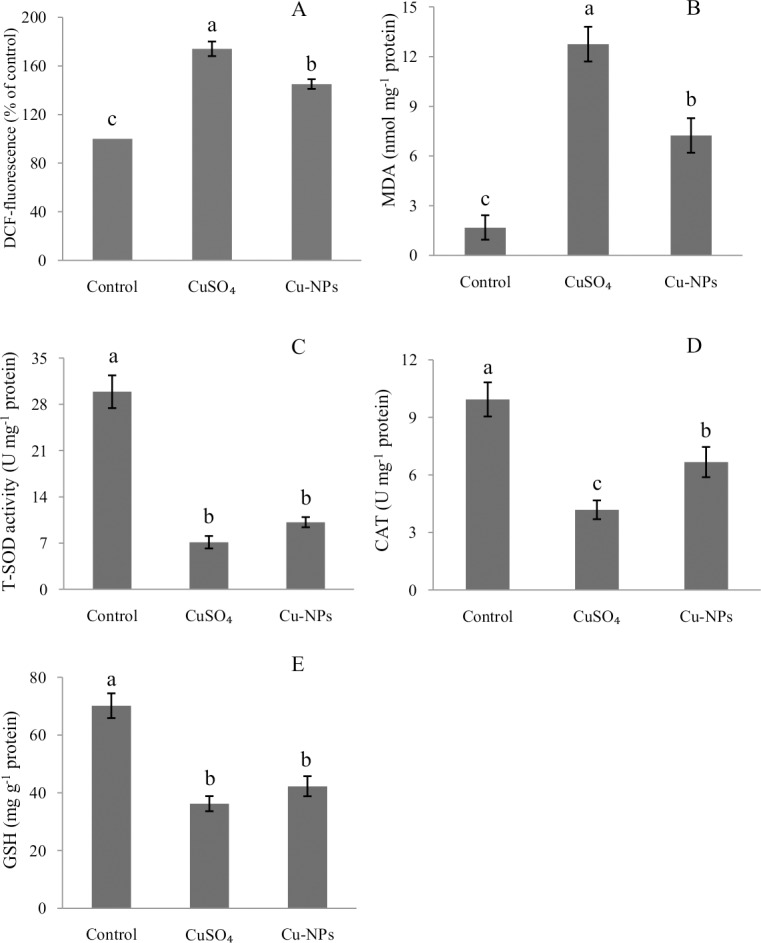
Reactive oxygen species (ROS) formation (A)and oxidative stress(B-E) in the primary hepatocytes of juvenile *E*.*coioides* after CuSO_4_ or Cu-NPs exposure. Data are means ±SD (n = 3). Significant differences (*p <*0.05) among treatments were indicated by different letters.

We measured ROS production using the fluorescent dye DCF-DA (Fig 3A). Compared to control, exposure to either CuSO_4_ or Cu-NPs significantly increased ROS generation(*p <*0.05),with CuSO_4_ resulting in significantly higher ROS than the equal concentration of Cu-NPs (*p <*0.05).

### Oxidative stress in hepatocytes

The changes in lipid peroxidation and antioxidant capacity are shown in Fig (3B–3E). Compared to control, either CuSO_4_ or Cu-NPs caused a significant increase in MDA formation (*p <*0.05), and significant decreases in the activities of T-SOD, CAT and GSH (*p <*0.05), more so in the CuSO_4_ than Cu-NPs treatment.

### Cell apoptosis and necrosis

**Fig 4:**
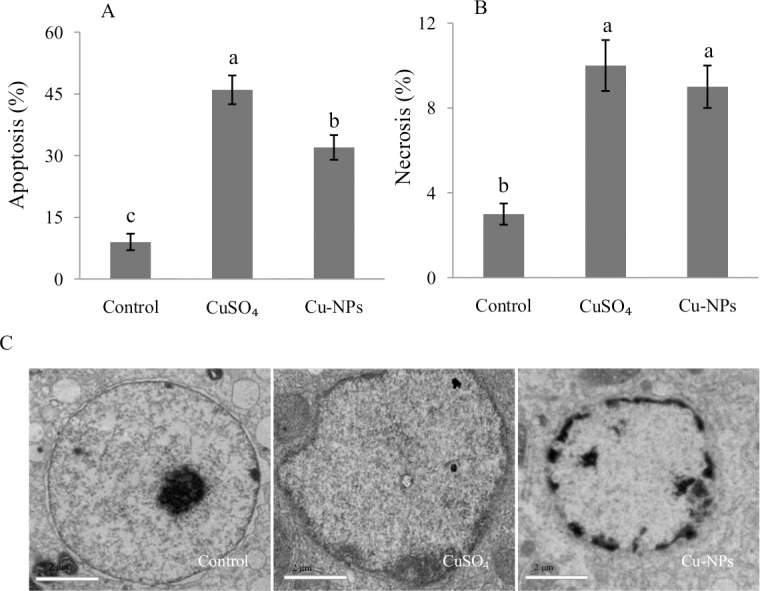
Cell apoptosis and necrosis in the primary hepatocytes of juvenile *E*.*coioides* after CuSO_4_ or Cu-NPs exposure. (A) cell apoptosis; (B) cell necrosis; (C) the nuclear ultrastructure examined by transmission electron microscopy (TEM), scale bar = 2 μm. Data are means ±SD (n = 3). Significant differences (*p <*0.05) among treatments were indicated by different letters.

The percent of apoptotic (Fig 4A) and necrotic hepatocytes (Fig 4B) was significantly higher after the CuSO_4_ or Cu-NPs treatment compared with the control (*p <*0.05), with CuSO_4_ causing higher apoptosis and necrosis than Cu-NPs. The ultrastructural alterations in apoptotic cells were observed under transmission electron microscopy (Fig 4C). The control cells displayed normal nuclear morphology. In contract, either CuSO_4_ or Cu-NPs treatment resulted in typical apoptotic features including chromatin condensation and margination at the nuclear periphery.

### Cytochrome c

**Fig 5:**
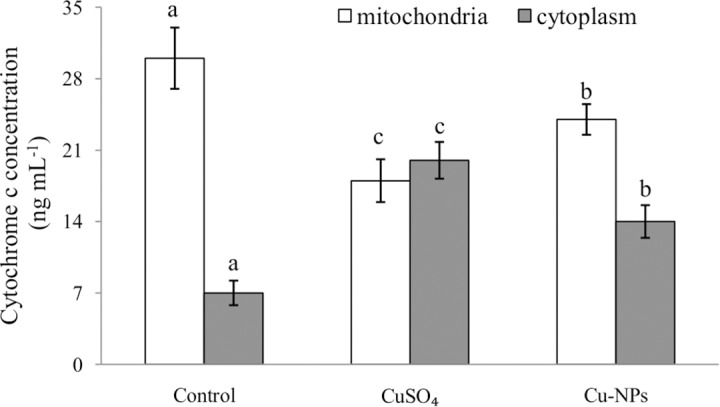
Cytochrome c concentration in primary hepatocytes of juvenile *E*.*coioides* after CuSO_4_ or Cu-NPs exposure. Data are means ±SD (n = 3). Different letters denoted results significantly different from control (*p <*0.05).

Compared to control, exposure to either CuSO_4_ or Cu-NPs induced a significant increase in cytochrome c concentrations in the cytosol and a significant decrease in mitochondria (*p <*0.05)(Fig 5), more so in the CuSO_4_ than Cu-NPs treatment.

### Caspase activities

**Fig 6:**
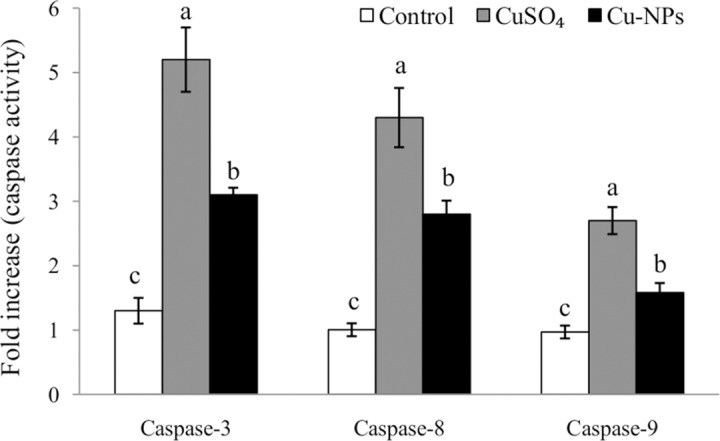
Caspases activities in primary hepatocytes of juvenile *E*.*coioides* after CuSO_4_ or Cu-NPs exposure. Data are means ±SD (n = 3). Significant differences (*p <*0.05) among treatments were indicated by different letters.

Apoptosis was evaluated by proteolytic activities of caspase-3, caspase-8 and caspase-9 (Fig 6). After CuSO_4_ or Cu-NPs exposure, significant increases in caspase-3, caspase-8 and caspase-9 activities were noticed compared to control (*p <*0.05), with the CuSO_4_ treatment resulting in significantly higher caspase-3, caspase-8 and caspase-9 activities than Cu-NPs (*p <*0.05).

### Gene expression

**Fig 7:**
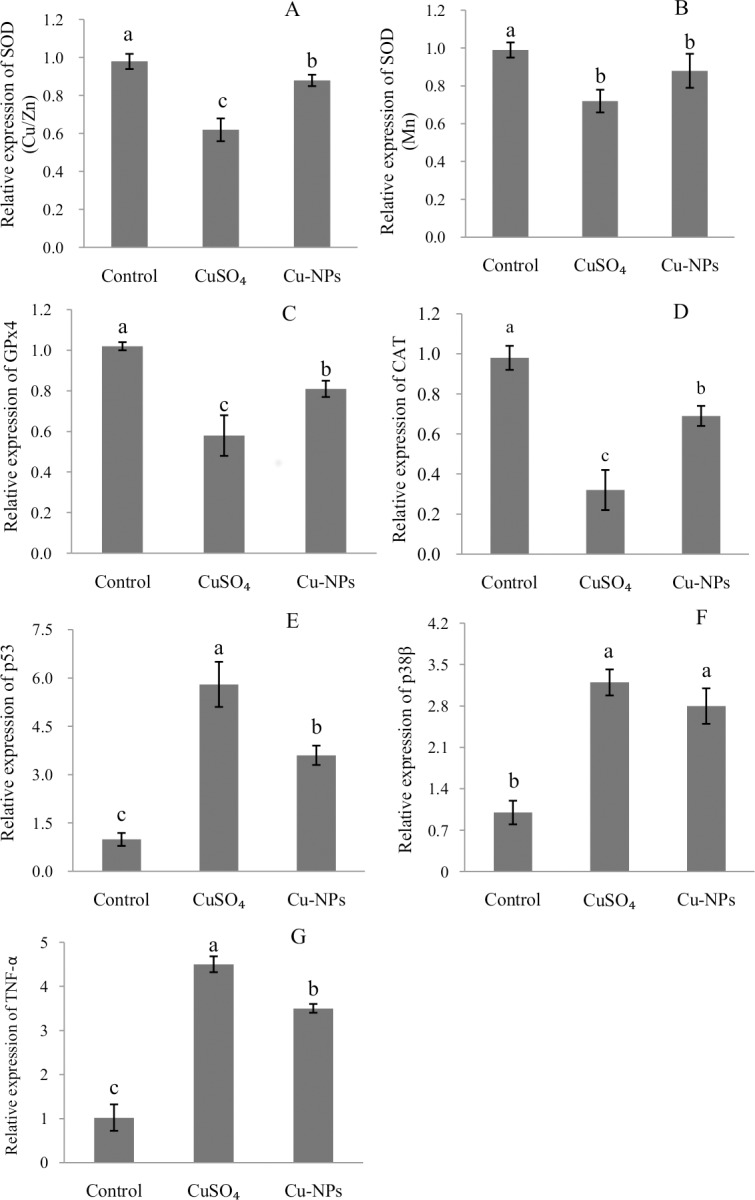
Anti-oxidative and apoptosis-related gene expressions in primary hepatocytes of juvenile *E*.*coioides* after CuSO_4_ or Cu-NPs exposure. Anti-oxidative related gene expression (A-D), apoptosis related gene expression(E-G). Data are means ±SD (n = 3). Significant differences (*p <*0.05) among treatments were indicated by different letters.

Compared to control, the antioxidative-related genes [*SOD (Cu/Zn)*, *SOD (Mn)*, *GPx4*, *CAT*] were significantly down-regulated. However, genes related to apoptosis (*p53*, p*38β* and *TNF-α*) were significantly up-regulated in hepatocytes than control after CuSO_4_ or Cu-NPs treatment (*p <*0.05) (Fig 7).

### Protein abundance

**Fig 8:**
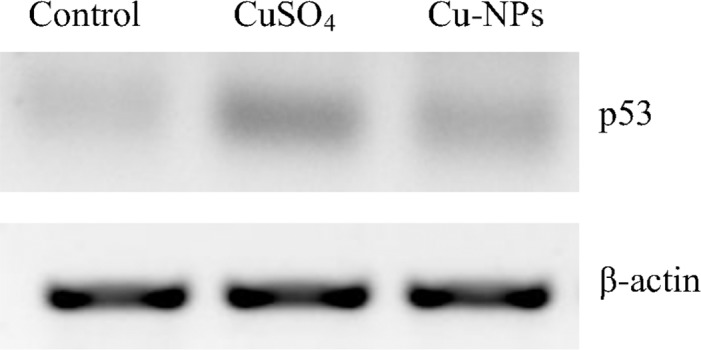
p53 protein expression in primary hepatocytes of juvenile *E*.*coioides* after CuSO_4_ or Cu-NPs exposure.

Compared to control, abundance of p53 protein was increased more in the CuSO_4_ than Cu-NPs treatment (Fig 8).

## Discussion

To our knowledge, this is the first study that focused on the effects of CuSO_4_ or Cu-NPs on hepatocyte primary cultures of *E*.*coioides*. The primary and secondary nanoparticle sizes are regarded as important parameters for *in vitro* cytotoxicity in a cell culture medium[17]; therefore, the behavior of Cu-NPs in cell culture medium was evaluated through nanoparticle tracking analysis (NTA) to understand the extent of aggregation and secondary size of these nanoparticles before cellular exposure. In the present study, the average diameter of Cu-NPs in the cell culture medium was larger (100 ±35 nm) than the primary particle diameter (80±32 nm), suggesting aggregation of nanoparticles. However, the average secondary particle diameter was smaller in the cell culture medium (100 ±35 nm) than seawater (210 ±130 nm) in our previous study [7], suggesting relatively less particle aggregation in the cell culture medium than seawater. These results were corroborated well by other reports [17,32].

In the present study, primary *E*.*coioides* hepatocyte cultures were used to demonstrate CuSO_4_ or Cu-NPs cytotoxicity as measured by the MTT and LDH assays. It is worth emphasizing that MTT assay reflects only the changes in mitochondrial function, and is not indicative of the manner or stages of cell death [24, 33]. Niska et al. [33] reported that a reduction in the amount of formazan produced from MTT can be proportional to the cell number. Cui et al. [27] reported LDH is a soluble cytosolic enzyme present in most eukaryotic cells that is released into the culture medium upon cell death due to plasma membrane damage. Hence, an increase in the LDH activity in culture supernatant is proportional to the number of lysed cells [34].In the present study, the proportion of viable cells declined after CuSO_4_ or Cu-NPs exposure compared to control. The question remained as to how CuSO_4_ or Cu-NPs induced cell death.

Evidence of hazardous health effect of nanoparticlesis increasing rapidly. ROS generation has been proposed as a possible mechanism involved in the toxicity of nanoparticles[24,35]. Valko et al. [36] and Paz-Elizur et al. [37] reported ROS are generally considered cytotoxic because the free radicals cause oxidative damage to biomolecules such as DNA, proteins and lipids through oxidative modifications. However, studies on the exact mechanisms by which nanoparticles generated ROS in cells are still underway [24]. In the present study, we found that CuSO_4_ or Cu-NPs increased cellular ROS generation. These results are in good agreement with Jia et al. [14] and Cui et al.[27]. We also observed an increase in MDA concentration (a marker of lipid peroxidation) and decreases in the activities of antioxidants including T-SOD, CAT and GSH following the CuSO_4_ or Cu-NPs treatment. In addition, the antioxidative-related genes [*SOD (Cu/Zn)*, *SOD (Mn)*, *CAT*, *GPx4*] were also down-regulated in hepatocytes, consistent with earlier findings that CuSO_4_ or Cu-NPs reduced T-SOD, CAT and GSH activities in whole-fish cultured in seawater [7]. Hence, we conclude that (1) toxicity to whole fish can be replicated in hepatocyte cultures; (2) Cu from CuSO_4_ or Cu-NPs stimulated the intracellular ROS generation, altered the antioxidative enzymatic defense systems in primary hepatocytes of *E*.*coioides* causing apoptosis and necrosis. Our study also found higher ROS generation, higher MDA concentration, lower activities of antioxidative enzymes T-SOD and CAT and lower concentration of antioxidant GSH in the CuSO_4_ than Cu-NPs treatment. However, Thit et al. [38] reported Cu-NPs were more toxic than soluble Cu to kidney epithelial cells from frog *Xenopuslaevis* (A6).The difference between the two studies could be due to different animal species; it should also be kept in mind that differential susceptibility to Cu could exist between freshwater and seawater species.

ROS are important for apoptosis [2,14].The mechanisms driving apoptosis in cells under oxidative stress may involve high ROS concentration directly inhibiting caspase (cysteine protease) activity, disrupting intracellular Ca^2+^ homeostasis, and leading to ATP (adenosine triphosphate) depletion [39]. In the present study, apoptosis and necrosisin hepatocytes were significantly higher after CuSO_4_ or Cu-NPs treatment than in control. Thus, oxidative stress likely plays an important role in apoptosis induced by CuSO_4_ or Cu-NPs in *E*.*coioides* hepatic cells.

Apoptosis is a highly organized and genetically-controlled type of cell death that occurs under a variety of physiological and pathological conditions [22,40].Although apoptosis can be triggered by various stimuli, there are two main apoptotic pathways: mitochondria-initiated intrinsic and death receptor-triggered extrinsic[41].Both pathways activate a set of caspases [14]. In general, caspase-3 is the major operational caspase, which plays an essential role in apoptosis by mediating a subsequent lethal chain of events [22].Caspase-8 and caspase-9 are the major initiator caspases implicated in the two pathways [42].

Many studies have demonstrated apoptosis via caspase-dependent pathway [14,18,22]. In the present study, a significant increase in the activities of caspase-3, caspase-8 and caspase-9 was noticed after CuSO_4_ or Cu-NPs exposure compared to control, indicating that CuSO_4_ or Cu-NPs participated in the activation of caspases to induce hepatocyte apoptosis. Our study also found the CuSO_4_ treatment induced higher caspase-3, caspase-8 and caspase-9 activities than the Cu-NPs treatment, suggesting caspases in the primary hepatocytes of *E*.*coioides* were more sensitive to Cu ions than Cu-NPs.

Several signaling pathways have been implicated in the initiation of the caspase cascade. One of the most well defined pathways for pro-caspase activation is the translocation of cytochrome c from mitochondria to the cytosol [43].The disruption of the mitochondrial membranes may allow cytochrome c leakage into the cytosol [44], with the released cytochrome c and deoxyadenosine triphosphate (dATP) binding to the apoptotic protease activating factor-1 (APAF-1), leading to the recruitment and activation of caspases[14,22].In the present study, we investigated a key event in the activation of caspases, the release of cytochrome c, and demonstrated that apoptosis in primary hepatocytes induced by CuSO_4_ or Cu-NPs was accompanied by enhanced release of cytochrome c into the cytoplasm. The mechanism of cytochrome c release from mitochondria is not well understood, but it may be associated with an interaction of ROS with mitochondria [45].

To clarify a possible mechanism of apoptosis, we investigated the effects of CuSO_4_ and Cu-NPs on apoptosis-related genes and p53 protein. The tumor-suppressor protein p53 is a universal sensor of environmental stress and, as a transcription factor, plays a critical role in regulating expression of genes involved in mediating growth arrest and/or apoptosis[30]. Ahamed et al. [24] reported p53 protein triggered a cell cycle arrest (to provide time for the damage to be repaired) or self-mediated apoptosis in the presence of DNA damage or cellular stress; consequently, p53 has been used as a sensitive biomarker in response to gene toxicity. In this study, gene expression and abundance of p53 increased in hepatocytes after CuSO_4_ or Cu-NPs treatment, with CuSO_4_being more effective than the equal concentration of Cu-NPs. Those results indicated that CuSO_4_ or Cu-NPs induced gene toxicity to primary *E*.*coioides* hepatocytes, and activation of p53 by genotoxic injury can result in either growth arrest or apoptosis. Qi et al. [30] found that transcription of p53 could be activated by increased ROS generation; indeed, ROS are considered a key factor in the activation of p53 by many chemotherapeutic agents and stimuli that may not be DNA-damaging agents [46].However, further studies are needed to elucidate pathways enabling ROS to influence abundance of protein p53.

The p38 mitogen-activated protein kinases (MAPKs) (including p38a, p38b and p38β) are the signaling molecules involved in regulation of cellular responses to various extracellular stimuli[47]. Cai et al.[15] reported p38β played a crucial role in regulating apoptosis. In the present study, we found p38β was up-regulated in hepatocytes after CuSO_4_ or Cu-NPs treatment, with CuSO_4_ resulting in higher p38β gene expression than Cu-NPs.

TNF-α, a pleiotropic cytokine, mediates metal-induced hepatotoxicity by inflammatory signaling pathways and stimulates inflammation and fibrosis; when protein synthesis is repressed, TNF-α turns into a death factor causing cells apoptosis [14,48]. Suska et al.[48] reported oxidative stress triggered the TNF-α release, and resulted in exacerbated apoptosis. Pan et al. [49] reported overexpressing of TNF-α could cause endotoxic shock and may contribute to fish disease. In our study, the gene expression of TNF-α was significantly up-regulated in hepatocytes after CuSO_4_ or Cu-NPs treatment compared to control, with CuSO_4_ exposure resulting in higher TNF-α expression than the equal concentration of Cu-NPs. These findings indicted either form of Cu may contribute to primary hepatocyte pathologies and exacerbated apoptosis (as judged from flow cytometry analysis), but Cu ions may cause more severe toxicity to primary hepatocytes than Cu-NPs.

## Conclusions

**Fig 9:**
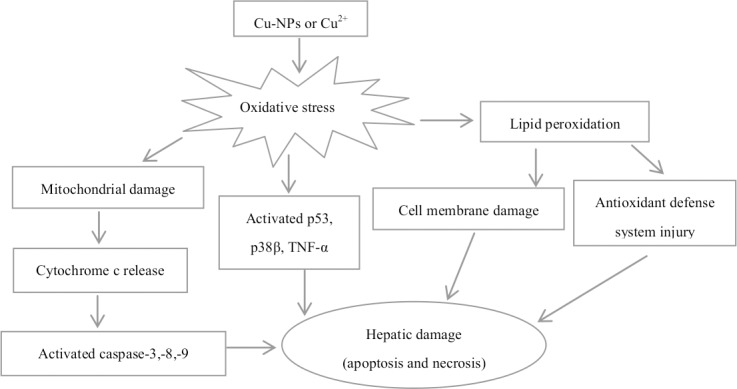
Scheme showing the proposed mechanisms of Cu-NPs and CuSO_4_ toxicity to primary hepatocytes of *E*.*coioides*. Cu-NPs and CuSO_4_ exhibited similar types of toxic mechanisms.

The present study demonstrated the mechanisms of Cu-NPs and CuSO_4_ toxicity to hepatocytes (Fig 9). Cu-NPs had similar types of toxic mechanisms as CuSO_4_ on primary hepatocytes of *E*.*coioides*. Exposure to either Cu-NPs or CuSO_4_ increased cellular ROS. These ROS could induce oxidative stress and lipid peroxidation, which impaired membrane structure and anti-oxidant defense system. In addition, increased ROS may impair mitochondrial bioenergetics and physiological functions. Subsequently, a release of mitochondrial cytochrome c into the cytosol activated caspases, triggering apoptosis. In addition, the apoptosis-related genes (*p53*, *p38β* and *TNF-α*) were activated by oxidative stress. Due to their cytotoxic effects, both CuSO_4_ and Cu-NPs should be strictly monitored in the *E*.*coioides* culture.
